# CECT-based simplified scoring model for differentiating low-risk thymomas from hyper-attenuating thymic cysts: A STARD compliant article

**DOI:** 10.1097/MD.0000000000044453

**Published:** 2025-09-05

**Authors:** Lin Zhao, Jingwu Li, Yongliang Liu, Wenzhe Zhao, Haifeng Cai, Lixiu Cao

**Affiliations:** a Department of Computed Tomography, Tangshan People’s Hospital, Tangshan, Hebei Province, China; b Hebei Key Laboratory of Molecular Oncology, Tangshan, Hebei Province, China; c Department of Neurosurgery, Tangshan People’s Hospital, Tangshan, Hebei Province, China; d Department of Breast Surgery, Tangshan People’s Hospital, Tangshan, Hebei Province, China; e Department of Nuclear Medicine Imaging, Tangshan People’s Hospital, Tangshan, Hebei Province, China.

**Keywords:** anterior mediastinal hyper-attenuating nodules, CECT, low-risk thymomas, machine learning, thymic cysts

## Abstract

This retrospective study aims to evaluate the effectiveness of a simplified scoring model utilizing contrast-enhanced computed tomography (CECT) in distinguishing low-risk thymomas (LRTs) from thymic cysts in patients with anterior mediastinal hyper-attenuating nodules. A total of 32 patients of LRTs and 40 patients of hyper-attenuating thymic cysts who underwent chest biphasic CECT preoperatively from January 2015 to December 2022 were included. The traditional CT imaging features and clinical features of each patient were analyzed. A predictive model was built by multivariable logistic regression, and subsequently, a simplified scoring model was developed according to the regression coefficients of each risk factor of LRTs. The performance of risk factors and models were assessed by receiver operating characteristic curve, decision curve analysis, and DeLong test. Compared to hyper-attenuating thymic cysts, LRTs tended to be located off-midline, higher CT values in the venous phase, and moderate to severe enhancement (all *P* < .001). Based on the above risk factors of LTRs, the predictive model achieved an area under the receiver operating characteristic curve of 0.938. While the simplified scoring model demonstrated comparable diagnostic ability (area under the receiver operating characteristic curve = 0.936, *P* = .42), with ideal sensitivity (0.719), accuracy (0.861), and specificity (0.975). Decision curve analysis indicated this scoring model provided a higher clinical net benefit. Biphasic CECT had a strong diagnostic capability in differentiating LRTs from hyper-attenuating thymic cysts. The diagnostic scoring model is straightforward and convenient, making it easy to popularize.

## 1. Introduction

Thymic epithelial tumors (TETs) are the most common primary tumors of the anterior mediastinum, accounting for 47% of mediastinal neoplasms.^[1,2]^ In 2004, TETs were categorized into 3 risk subgroups based on increasing malignancy grade: low-risk thymomas (LRTs: types A, AB, and B1), high-risk thymomas (types B2 and B3), and thymic carcinoma.^[3]^ Although LRTs exhibits less invasive behavior and higher survival rates compared to high-risk thymomas, active surgical management is still warranted for LRTs due to the unpredictability of clinical behavior, even in asymptomatic patients.^[4–8]^ Thymic cysts are benign and rare lesions that primarily occur in the anterior mediastinum, representing 1% to 3% of all mediastinal masses.^[9,10]^ When a thymic cyst contains high protein levels or is accompanied by bleeding, it appears hyper-attenuating (CT values in the unenhanced phase [CTU] > 20 HU), resembling solid density. In some cases, it is challenging to differentiate hyper-attenuating thymic cysts from thymic solid tumors using traditional imaging examinations, especially cysts from LRTs.^[10–12]^ However, there are significant differences in the biological properties, treatments, and surgical approaches between LRTs and thymic cysts.^[10,13]^ Furthermore, biopsy carries certain risks due to the proximity of anterior mediastinal lesions to the heart and major mediastinal vessels.^[14,15]^ Therefore, accurately and noninvasively differentiating LRTs from thymic cysts through traditional examinations prior to treatment is crucial for the selection of individualized treatment strategies.

There is a consensus that chest CT, particularly contrast enhanced computed tomography (CECT), remains the preferred, most reliable, and, cost-effective examination method for thymic tumors, as outlined in the National Comprehensive Cancer Network guidelines.^[16]^ However, challenges arise in differentiating hyper-attenuating thymic cysts from LRTs due to overlapping imaging features. For instance, Wang et al reported that although thymic cysts were benign in terms of biological characteristics, the CT imaging features of certain cysts with a diameter of ≤3 cm and an unenhanced CT value >20 HU resembled those of thymomas, particularly LRTs.^[10]^ Furthermore, many patients undergo invasive surgery and related complications due to the misinterpreting thymic cysts as thymomas on chest CT. According to previous reports, the rate of unnecessary thymectomy is as high as 22% to 44%.^[17,18]^ To our knowledge, no previous study has established comprehensive differential diagnostic criteria using machine learning and existing chest CECT images to effectively characterize LRTs from thymic cysts in patients with anterior mediastinal hyper-attenuating (CTU > 20 HU) nodules (long diameter [LD] ≤ 3 cm).

This study aimed to explore whether a simplified scoring model based on chest biphasic CECT could effectively distinguish thymic cysts from LRTs in patients with anterior mediastinal hyper-attenuating nodules. This differentiation may facilitate a greater number of patients receiving less or noninvasive diagnostic approaches, ultimately contributing to a reduction in the rate of nontherapeutic thymectomy.

## 2. Materials and methods

### 2.1. Patients

**Figure 1.**
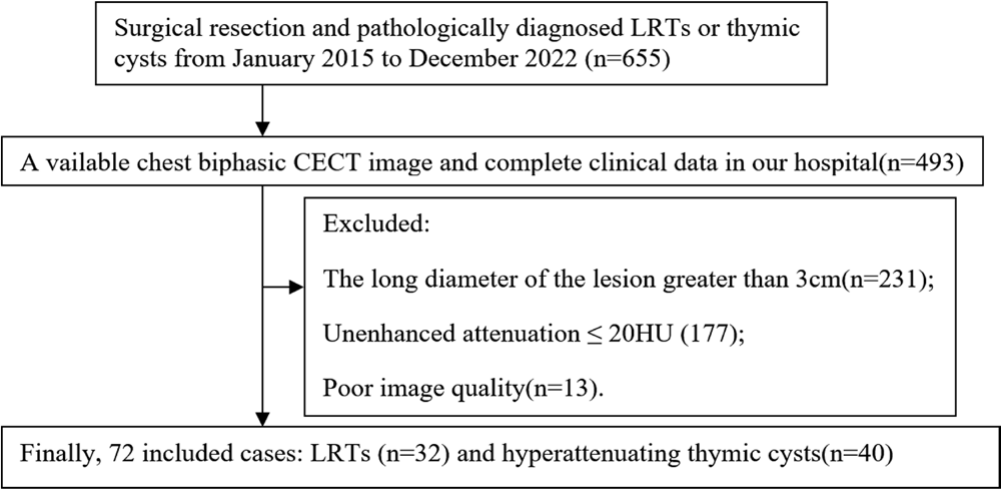
The flow diagram of sample selecting.

This retrospective study was approved by Tangshan People’s Hospital Institutional Ethics Committee (No. RMYY-LLKS-2024076), and this study was conducted in accordance with the Declaration of Helsinki (Version 2013). Informed consent was obtained from each patient. Inclusion of patients who met the following inclusion criteria between January 2015 and December 2022: patients who underwent surgical resection and received a pathological diagnosis of LRTs or thymic cysts; patients who had a chest biphasic CECT scan performed within 2 weeks prior to surgery and had not received any previous treatment; patients with anterior mediastinal hyper-attenuating (CTU > 20 HU) nodules (LD ≤ 3 cm); patients with complete imaging and clinical information; and patients with good image quality. Ultimately, a total of 32 LRTs and 40 thymic cysts were included in our study (see Fig. 1). The age, gender, and clinical symptoms of both groups were recorded and compared.

### 2.2. Image protocol

The GE Discovery CT750 HD (GE Healthcare, Boston) and the Ingenuity Core 64 (Philips Healthcare, the Netherlands) were utilized in this retrospective analysis. All patients underwent chest unenhanced as well as CECT scans, which included an arterial phase (approximately 23 seconds) and a venous phase (approximately 56 seconds). This was conducted following the infusion of 100 mL of the nonionic contrast agent iodopamil at a rate of 3 mL/s using a high-pressure syringe. The scanning and reconstruction parameters are detailed in Table 1.

**Table 1:** The scanning parameters and image reconstruction for chest biphasic CECT scan.

	GE 64-MDCT chest biphasic CECT scan	Philips 64-MDCT chest biphasic CECT scan
Tube current	Automated tube current	Automated tube current
Tube voltage	120 kV	120 kV
Reconstruction algorithm	Soft tissue standard algorithm	Soft tissue standard algorithm
Slice thickness	5 mm	2 mm
Slice increment	5 mm	2 mm
Reconstructed thickness	1.25 mm	
Reconstructed thickness	1.25 mm	
Reconstruction matrix	512 × 512	512 × 512
Number of cases	57	15

CECT = contrast enhanced computed tomography.

### 2.3. Imaging analysis

Two radiologists who were unaware of the pathological results and clinical information independently measured and evaluated the imaging features of lesions on thin-sliced CT images. Their experience in chest CT diagnosis spanned 6 and 10 years, respectively. The evaluation included various characteristics of the lesions, such as morphology (round or oval was classified as regular and all other shapes as irregular), border (categorized as well-defined or not well-defined), location (the epicenter of the lesion located directly behind the sternum was defined as midline, otherwise off-midline), LD, short diameter, attenuation (classified as homogeneous or heterogeneous), calcifications, CTU, CT values in the arterial phase (CTA), CT values in the venous phase (CTV), the presence of cystic or necrotic tissue (defined as low-density regions without enhancement), and the presence of mass effect. Invasiveness was evaluated based on involvement of the pleura, chest wall, vascular structures, or pericardium. Any discrepancies in data were resolved by consensus. Measure CT values using a region of interest defined as two-thirds of the largest cross-section of the nodule, while avoiding artifacts, calcifications, cystic and necrotic areas, as well as the periphery of the lesions. Furthermore, we measured CT values 3 times and recorded the average as the final result. The degree of enhancement was defined as mild to moderate or moderate to severe based on whether the CT value increased by <25 HU or more than 25 HU, respectively.^[19]^ If the difference in enhancement level between arterial and venous phases was <5 HU, it was considered as equal enhancement; otherwise, the phase in which the maximum enhancement level was defined as the peak enhancement phase.^[20]^

### 2.4. Statistical analysis and predictive model

R software (version 4.4.2, Lucent Technologies, New Providence) was used to analyze all data. The Student *t* test or Mann–Whitney *U* test was employed to compare continuous variables, while the Fisher test or Chi-square test was used to compare categorical variables. receiver operating characteristic (ROC) curve was used to analyze all statistically significant variables, and the optimal cutoff values for quantitative variables were determined using the Youden index to maximize sensitivity and specificity. Then, independent risk factors for diagnosing LRTs were identified through multivariate logistic regression analysis, and a predictive model was built. Subsequently, a simplified scoring model was developed according to the regression coefficients of each independent risk factor of LRTs. The performance of risk factors and models were assessed by ROC curve and the area under the receiver operating characteristic curves (AUCs) were compared using DeLong test. The clinical value of risk factors and models were evaluated by decision curve analysis. *P* values < .05 were treated as significant.

## 3. Results

### 3.1. Pathological characteristics of LRTs and thymic cysts

According to the latest World Health Organization classification of thymic epithelial tumors (5th edition, 2021), types A, AB, and B1 thymomas are classified as LRTs.^[21]^ Type A thymoma is predominantly composed of thymic epithelial cells exhibiting spindle or oval nuclei, mild cytological atypia, finely granular chromatin, and inconspicuous nucleoli. These cells are typically arranged in sheets, fascicles, whorls, or rosette-like structures. Immature lymphocytes are sparse or absent, comprising <10% of the tumor area.^[21]^ Type AB thymoma consists of an admixture of lymphocyte-poor type A areas and lymphocyte-rich type B-like areas. The proportion of these components varies; they may be intermingled or sharply demarcated. Tumors composed solely of type A elements but containing moderate lymphocytes in over 10% of the area are also classified as type AB.^[21]^ Type B1 thymoma is characterized by scattered epithelial tumor cells embedded within a dense population of lymphocytes. The lymphocyte nuclei stain intensely basophilic (blue-purple) with hematoxylin; therefore, B1 thymomas often appear predominantly blue-purple on hematoxylin and eosin stained sections.^[21]^ Thymic cysts are characterized by a cyst lining composed of a single layer of flat, cuboidal, columnar, or squamous epithelium, with identifiable thymic tissue (including Hassall corpuscles and/or lymphocytes) within the cyst wall.^[21]^

Based on these histopathological diagnostic criteria, this study included 32 cases of LRTs (type A, n = 6; type AB, n = 21; type B1, n = 5) and 40 cases of thymic cysts.

### 3.2. The comparison of demographic characteristics and CECT imaging features

Patients with hyper-attenuating thymic cysts exhibited a higher percentage of males compared to those with LRTs (60.00% vs 34.38%, *P* = .03). Additionally, 87.50% of LRT cases were located off-midline, whereas only 50.00% of thymic cysts were found in similar locations (*P* < .001). Although the difference in CTU was statistically significant (*P = *.04), the average CTU of hyper-attenuating thymic cysts was only slightly lower than that of LRTs (37.43 vs 42.91 Hu). In contrast, both CTA and CTV values for LRTs were significantly higher than those for hyper-attenuating thymic cysts (all *P* < .001). Furthermore, after the enhanced scan, only mild to moderate enhancement of thymic cysts was observed, while LRTs demonstrated significant (moderate to severe) enhancement (*P* < .001), with nearly half of LRT cases showing cystic or necrotic features (*P* < .001). Notably, 78.13% of LRT cases were in the venous phase at peak enhancement levels, compared to only 32.50% of thymic cysts. However, 50.00% of thymic cysts exhibited equal enhancement, which was higher than LRTs. Overall, the peak enhancement phase showed a significant difference between LRTs and thymic cysts (*P* < .001). There were no significant differences in age, morphology, border, short diameter, LD, attenuation, calcifications, clinical symptoms, and the presence of mass effect between LRTs and hyper-attenuating thymic cysts (all *P* > .05) (Table 2).

**Table 2:** Comparison of demographic characteristics and CECT imaging features between LRT and thymic cysts.

Characteristics	LRTs (n = 32)	Thymic cysts (n = 40)	*P* value
Age	49.50 (41.75–58.25)	52.00 (42.00–65.25)	.32
Gender			**.03**
Male	11 (34.38%)	24 (60.00%)	
Female	21 (65.62%)	16 (40.00%)	
Morphology			.09
Regular	17 (53.13%)	29 (72.50%)	
Irregular	15 (46.87%)	11 (27.50%)	
Border			.29
Well-defined	25 (78.13%)	35 (87.50%)	
Not well-defined	7 (21.87%)	5 (12.50%)	
Location			**<.001**
Midline	4 (12.50%)	20 (50.00%)	
Off-midline	28 (87.50%)	20 (50.00%)	
LD	2.35 (1.78–2.90)	2.40 (1.97–2.90)	.43
SD	1.65 (1.38–2.23)	2.15 (1.60–2.50)	.14
Attenuation			.17
Homogeneous	22 (68.75%)	34 (85.00%)	
Heterogeneous	10 (31.25%)	6 (15.00%)	
Calcification			.17
Yes	8 (25.00%)	5 (12.50%)	
No	24 (75.00%)	35 (87.50%)	
CTU	**42.91 + 7.31**	**37.43 + 11.31**	**.04**
CTA	**64.44 + 12.92**	**49.08 + 13.47**	**<.001**
CTV	**71.25 + 11.79**	**51.23 + 13.29**	**<.001**
Cystic/necrotic			**.003**
Yes	17 (53.13%)	7 (17.50%)	
No	15 (46.87%)	33 (82.50%)	
Enhancement degree			**<.001**
Mild-moderate	14 (43.75%)	38 (95.00%)	
Moderate-severe	18 (56.25%)	2 (5.00%)	
Peak enhancement phase			**<.001**
Arterial phase	2 (6.25%)	7 (17.50%)	
Venous phase	25 (78.13%)	13 (32.50%)	
Equally enhanced	5 (15.62%)	20 (50.00%)	
Clinical symptoms			.92
Yes	6 (18.75%)	9 (22.50%)	
Cough	4 (12.50%)	5 (12.50%)	
Pain	2 (6.25%)	4 (10.00%)	
No	26 (81.25%)	31 (77.50%)	
Presence of mass effect			.96
Yes	2 (6.25%)	1 (2.50%)	
No	30 (93.75%)	39 (97.50%)	

Statistically significant variables were bolded.

CECT = contrast enhanced computed tomography, CTA = CT values in the arterial phase, CTU = CT values in the unenhanced phase, CTV = CT values in the venous phase, LD = long diameter, LRTs = low-risk thymomas, SD = short diameter.

**Figure 2.**
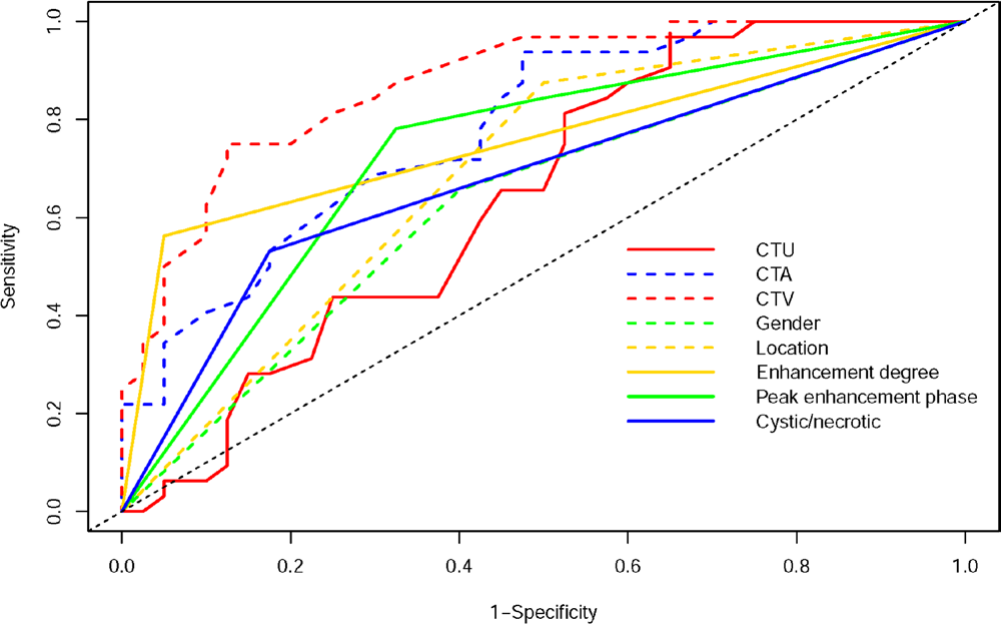
The ROCs of all the significant individual variables. ROC = receiver operating characteristic.

The AUC values of variable parameters that demonstrated statistical significance were derived through ROC curve. Among these variables, the AUC of CTV was greater than that of gender, location, CTU, CTA, enhancement degree, peak enhancement phase, and cystic/necrotic (see Table 3, Fig. 2). The cutoff values for CTU, CTA, and CTV were 31.0 HU, 50.5 HU, and 64.0 HU, respectively (refer to Table 3).

**Table 3:** Individual variables obtained from ROC analysis for differentiation of LRTs from thymic cysts.

Variables	Cutoff	AUC (95% CI)	Sensitivity	Specificity	Accuracy
CTU	31.00	0.644 (0.516–0.771)	96.9%	35.0%	62.5%
CTA	50.50	0.782 (0.678–0.886)	93.8%	52.5%	70.8%
CTV	**64.00**	**0.875 (0.796–0.954**)	**75.0%**	**87.5%**	**81.9%**
Gender	–	0.628 (0.515–0.742)	65.6%	60.0%	62.5%
Location	–	0.688 (0.553–0.822)	87.5%	50.0%	66.7%
Enhancement degree	–	0.756 (0.637–0.876)	56.2%	95.0%	77.8%
Peak enhancement phase phaphaephase phphase phase	–	0.730 (0.624–0.837)	78.1%	67.5%	72.2%
Cystic/necrotic	–	0.678 (0.533–0.823)	53.1%	82.5%	69.4%

Among the individual variables, the AUC value of CTV was the highest and has been marked in bold.

AUC = area under the receiver operating characteristic curve, CI = confidence interval, CTA = CT values in the arterial phase, CTU = CT values in the unenhanced phase, CTV = CT values in the venous phase, LRTs = low-risk thymomas, ROC = receiver operating characteristic.

### 3.3. Predictive model

**Figure 3.**
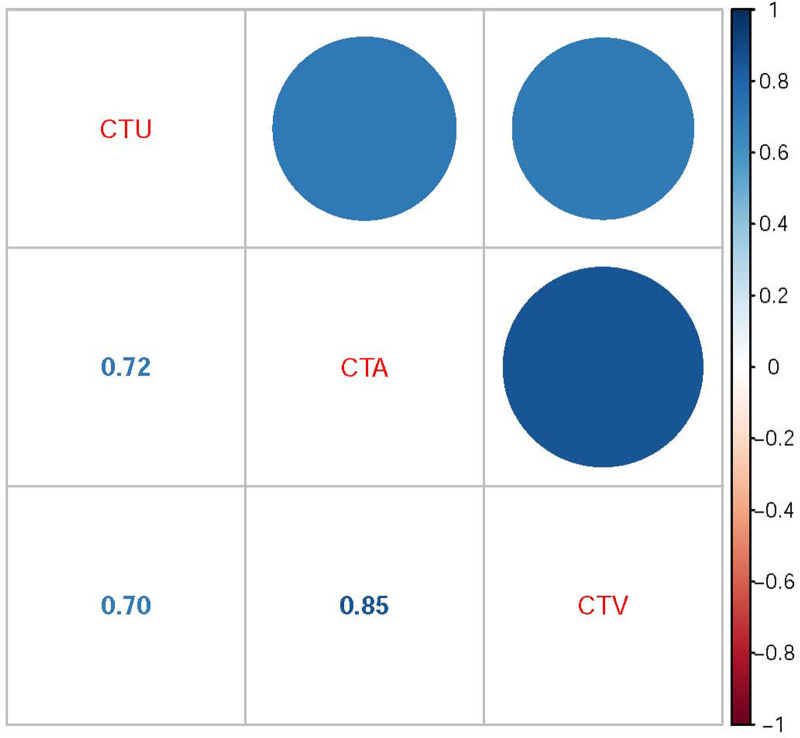
Strong correlations were observed between CTU, CTA, and CTV. CTA = CT values in the arterial phase, CTU = CT values in the unenhanced phase, CTV = CT values in the venous phase.

**Figure 4.**
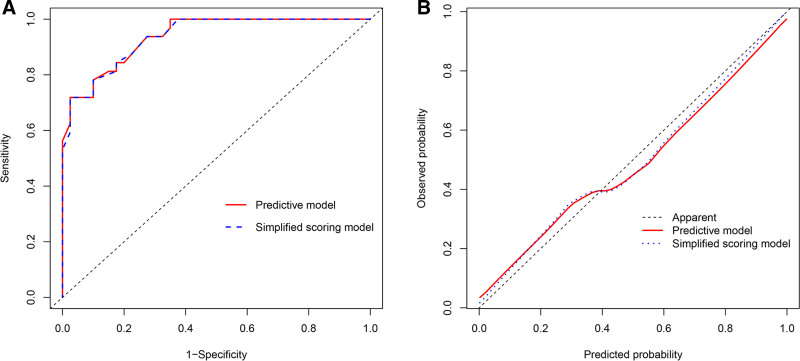
(A) The ROCs of the predictive model and the simplified scoring model; (B) good calibrations of the predictive model and the simplified scoring model were shown. ROC = receiver operating characteristic.

**Figure 5.**
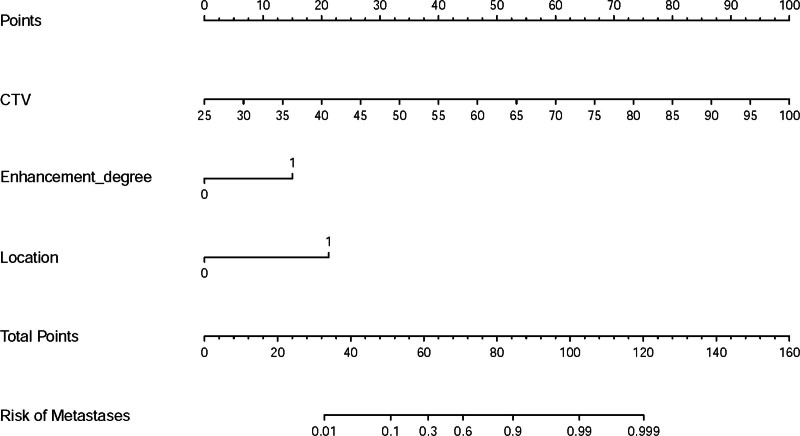
Nomogram of the predictive model.

Due to the strong correlations between CTU, CTA, and CTV, only CTV was selected for further analysis to reduce redundancies (Fig. 3). Subsequently, CTV, gender, peak enhancement phase, enhancement degree, location, and cystic/necrotic features were further analyzed by multivariate logistic regression. Finally, CTV, location, and enhancement degree were identified as independent risk factors for differential diagnosis of LRTs. Hyper-attenuating nodules located off-midline (odds ratio [OR], 16.341 [95% confidence interval [CI], 2.316–115.279]) with CTV > 64 HU (OR, 1.192 [95% CI, 1.068–1.331]) and moderate to severe enhancement (OR, 7.235 [95% CI, 0.697–75.113]) indicated a tendency towards LRTs. The predictive model demonstrated a high AUC value of 0.938 (95% CI, 0.888–0.987) with ideal specificity (97.5%), sensitivity (71.9%), and accuracy (86.1%) (Fig. 4A). The AUC of the predictive model was significantly higher than that of each independent risk factor (all *P* < .05, Table 4). The nomogram showed that a total score exceeding 74.74 could be considered as an indicator of LRTs (Fig. 5). A good calibration curve was also demonstrated (Fig. 4B).

**Table 4:** The comparison of the AUCs between the predictive model and independent risk factors.

Comparison	AUC	*Z* statistic	*P*
Predictive model vs CTV	0.938 vs 0.875	2.015	.04
Predictive model vs enhancement degree	0.938 vs 0.756	4.380	<.001
Predictive model vs location	0.938 vs 0.688	5.249	<.001

AUC = area under the receiver operating characteristic curve, CTV = CT values in the venous phase.

The regression coefficients for each risk factor in the predictive model were utilized to construct a scoring model, defined as follows:


$$\begin{array}{ll} The\;scoring\;model= & \;0.18\;*\;CTV\;\\ & +1.98\;*\;enhancement\;degree\;\\ & +2.97\;*\;location. \end{array}$$

To enhance usability, we further simplified the scoring model to:


$$\begin{array}{ll} The\;simplif\;ied\;scoring\;model= & \;0.2\;*\;CTV\;\\ & +2\;*\;enhancement\;degree\;\\ & +3\;*\;location. \end{array}$$

**Figure 6.**
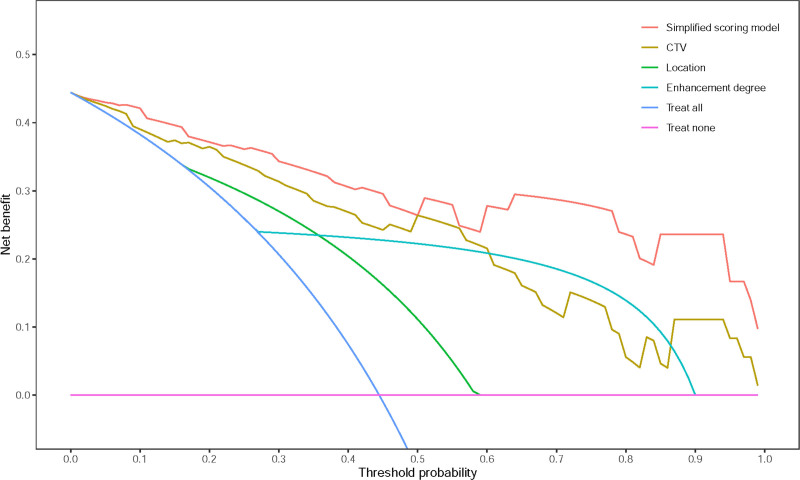
The DCAs of the simplified scoring model and independent risk factors. DCA = decision curve analysis.

The AUC of the simplified scoring model was 0.936 (95% CI: 0.886–0.986), which also demonstrated ideal sensitivity (71.9%), specificity (97.5%), and accuracy (86.1%) (Fig. 4A). According to the DeLong test, there was no significant difference in AUC between the 2 models (*P* = .42), indicating that simplifying regression coefficient did not affect the performance of the diagnostic models. The simplified scoring system also exhibited good calibration (Fig. 4B). In comparison to any independent risk factor, the decision curve analysis of the simplified scoring model provided the highest clinical net benefit across nearly all threshold probabilities, suggesting that this model is a promising tool for effectively distinguishing thymic cysts and LRTs in patients with anterior mediastinal hyper-attenuating nodules (Fig. 6).

**Figure 7.**
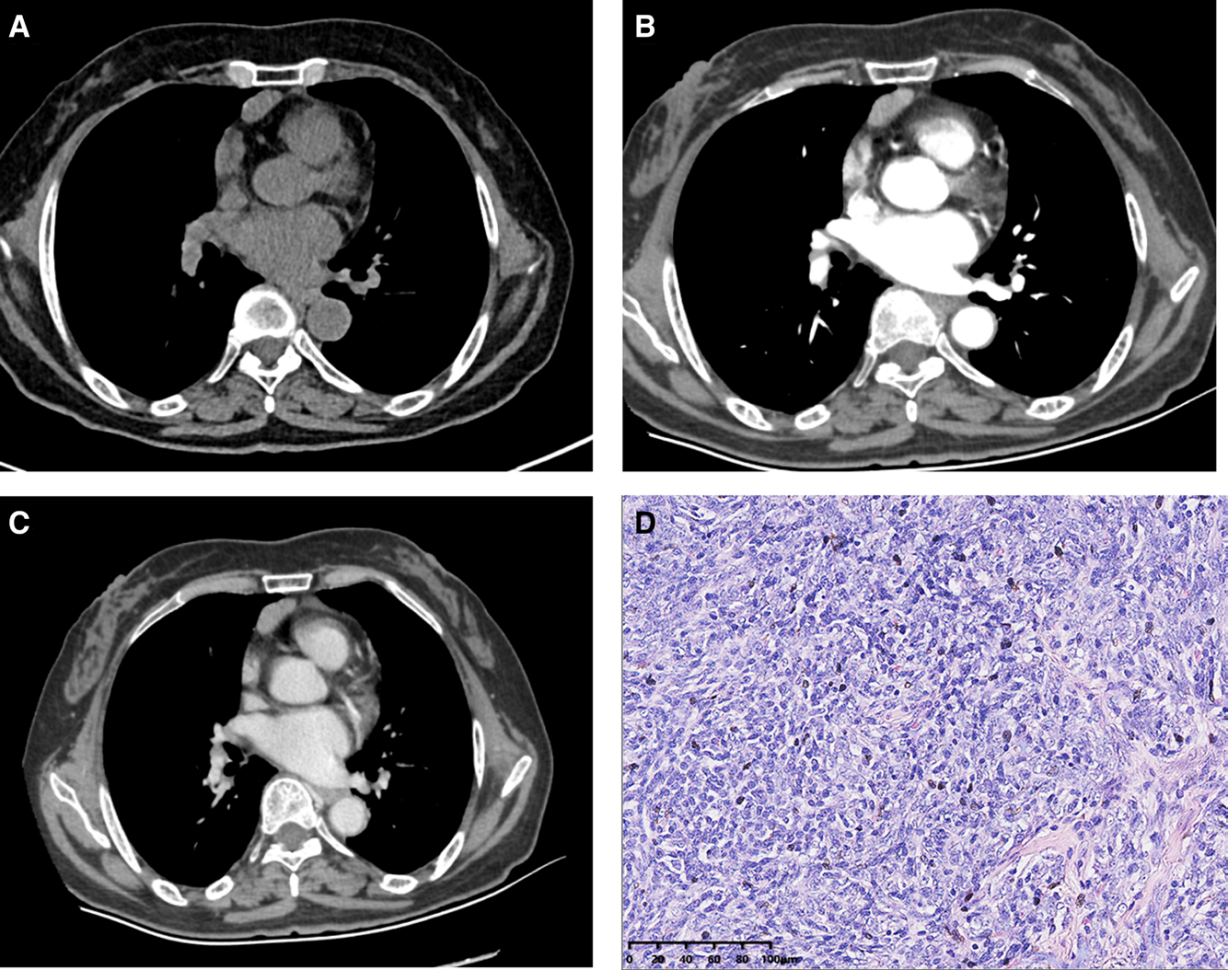
Low-risk thymoma. Anterior mediastinal nodule was found in a 69-year-old woman, which was off-midline location. The attenuation values on unenhanced (A), arterial (B), and venous (C) phases were 46, 93, and 91 HU, respectively. Moderate-severe (△CT = 45 HU ≥ 25 HU) enhancement. The nodule got a score of 23.2 points, indicating diagnosis of low-risk thymoma. (D) Histopathological features of low-risk thymoma (H&E staining, 20×). Scale bar: 100 μm.

**Figure 8.**
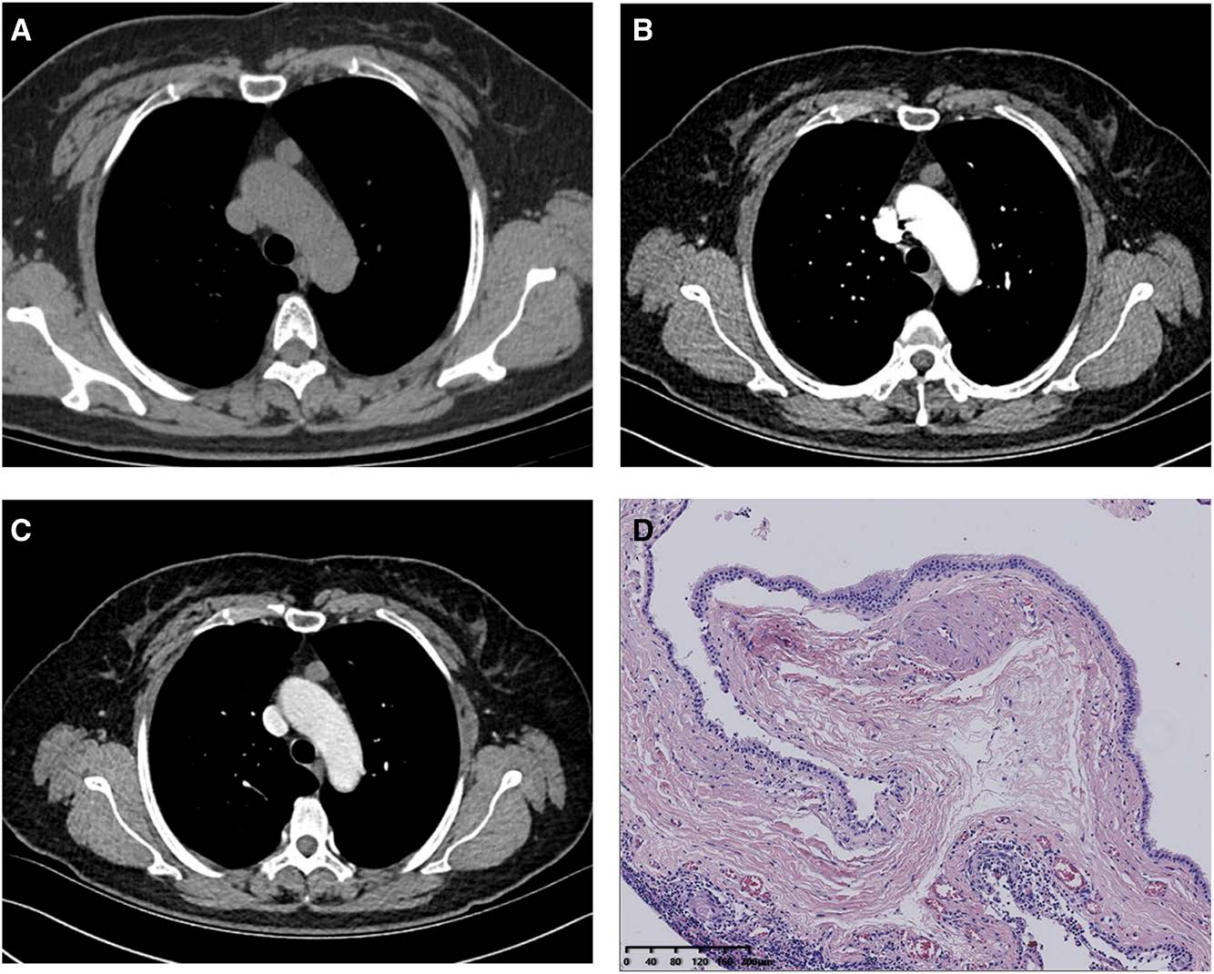
Thymic cyst. Anterior mediastinal nodule was found in a 54-year-old woman, which was off-midline location. The attenuation values on unenhanced (A), arterial (B), and venous (C) phases were 23, 42, and 46 HU, respectively. Mild-moderate (△CT = 23 HU < 25 HU) enhancement. The nodule got a score of 12.2 points, indicating diagnosis of hyperattenuating thymic cyst. (D) Histopathological features of thymic cyst (H&E staining, 10×). Scale bar: 200 μm.

The simplified score was determined by adding 2 points if the enhancement degree was classified as moderate to severe, 0 points for mild to moderate enhancement, and 20% of the CTV, and 3 points if the location was off-midline, 0 points for midline. The simplified scores for patients ranged from 6.6 to 24.2 points. The optimal cutoff value was 16 points, which demonstrated a specificity of 97.5% and a sensitivity of 71.9% (Table 5). This finding indicated that 71.9% of LRTs scores were ≥16, while 28.1% of LRT cases would be missed at this cutoff value. To minimize the rate of missed diagnoses, lower cutoff values may be considered. For instance, setting the cutoff at 13 could reduce the missed diagnosis rate to as low as 3.1%, albeit at the expense of specificity (65.0%) and precision (79.2%). Overall, higher scores from this simplified scoring system correlate with an increased risk of the lesion being classified as LRTs. Examples are provided in Figures 7 and 8.

**Table 5:** Diagnostic performance of the diagnostic score model with different cutoffs for LRTs.

Cutoff	Sensitivity (95% CI)	Specificity (95% CI)	Accuracy (95% CI)	PPV (95% CI)	NPV (95% CI)
10	1.000 (1.000, 1.000)	0.200 (0.076, 0.324)	0.556 (0.549, 0.562)	0.500 (0.378, 0.623)	1.000 (1.000, 1.000)
11	1.000 (1.000, 1.000)	0.375 (0.225, 0.525)	0.653 (0.647, 0.659)	0.561 (0.433, 0.690)	1.000 (1.000, 1.000)
12	1.000 (1.000, 1.000)	0.550 (0.396, 0.704)	0.750 (0.745, 0.755)	0.640 (0.507, 0.773)	1.000 (1.000, 1.000)
13	**0.969 (0.908, 1.000**)	**0.650 (0.502, 0.798**)	**0.792 (0.787, 0.796**)	**0.689 (0.554, 0.824**)	**0.963 (0.892, 1.000**)
14	0.938 (0.854, 1.000)	0.725 (0.587, 0.863)	0.819 (0.815, 0.823)	0.732 (0.596, 0.867)	0.935 (0.849, 1.000)
15	0.812 (0.677, 0.948)	0.825 (0.707, 0.943)	0.819 (0.815, 0.823)	0.788 (0.648, 0.927)	0.846 (0.733, 0.959)
16	**0.719 (0.563, 0.875**)	**0.975 (0.927, 1.000**)	**0.861 (0.858, 0.864**)	**0.958 (0.878, 1.000**)	**0.812 (0.702, 0.923**)
17	0.531 (0.358, 0.704)	1.000 (1.000, 1.000)	0.792 (0.787, 0.796)	1.000 (1.000, 1.000)	0.727 (0.610, 0.845)
18	0.531 (0.358, 0.704)	1.000 (1.000, 1.000)	0.792 (0.787, 0.796)	1.000 (1.000, 1.000)	0.727 (0.610, 0.845)
19	0.375 (0.207, 0.543)	1.000 (1.000, 1.000)	0.722 (0.717, 0.728)	1.000 (1.000, 1.000)	0.667 (0.547, 0.786)
20	0.219 (0.076, 0.362)	1.000 (1.000, 1.000)	0.653 (0.647, 0.659)	1.000 (1.000, 1.000)	0.615 (0.497, 0.734)

The optimal cutoff value was bolded.

CI = confidence interval, LRTs = low-risk thymomas, NPV = negative predictive value, PPV = positive predictive value.

## 4. Discussion

As is well known, when the density of cyst fluid increases or the cyst fluid becomes viscous, thymic cysts may present as hyper-attenuating, defined as a plain CT measurement value >20 Hu, which corresponds to solid density in conventional definitions. This phenomenon results in low preoperative diagnostic accuracy for hyper-attenuating thymic cysts and may lead to unnecessary surgical interventions for patients.^[10,12,17,22,23]^ Furthermore, the morphology of small thymomas (with a LD ≤ 3 cm), particularly LRTs, often resembles that of thymic cysts, thereby increasing the misdiagnosis rate.^[24]^ Accurate differentiation between LRTs and thymic cysts is crucial for effective treatment planning in patients with anterior mediastinal hyper-attenuating nodules. However, due to the limitations associated with percutaneous biopsy in the anterior mediastinum, imaging examinations, especially conventional CECT, serve as the primary basis for preoperative diagnosis.^[25–27]^ In our study, the diagnostic model developed using traditional CECT imaging features (including CTV, location, and enhancement degree) demonstrated robust performance in effectively distinguishing between the 2 groups. Additionally, the simplified scoring model exhibited comparable diagnostic value to the predictive model and held significant promise for clinical application due to its simplicity and convenience.

In our study, the CTU, CTA, and CTV values were all significantly higher for LRTs compared to hyper-attenuating thymic cysts (*P* < .05). Given the strong correlations observed among CTU, CTA, and CTV, we selected CTV as the independent factor for differentiating LRTs from thymic cysts based on the AUC value. The highest AUC of CTV in our study suggested that the enhanced CT value could serve as a basis for distinguishing between thymic cysts and LRTs, which was consistent with the research results of Jin et al.^[12]^ However, the AUC, sensitivity, and specificity of CTV in differentiating LRTs from hyper-attenuating thymic cysts in our study were 0.875, 75.0%, and 87.5%, respectively. These values were lower than those reported by Jin et al,^[12]^ who found an AUC of 0.950, with a specificity of 95.65% and a sensitivity of 88.89% for the differential diagnosis of thymoma and thymic cysts. But their sample size (n = 40) was smaller than that of our study. Additionally, Ackman et al^[11]^ found that the overall diagnostic sensitivity for patients with cysts with an unenhanced CT value >20 Hu and a diameter ≤ 3 cm was only 79.6% and 23.1%, respectively. It is noteworthy that the differential diagnosis of LRTs and hyper-attenuating thymic cysts has consistently posed challenges in daily clinical and imaging practice, and there are currently few reports addressing this specific research topic. Therefore, the inconsistent results across studies highlight the need for further verification through large-sample research.

The location and enhancement degree of anterior mediastinal hyper-attenuating nodules were also identified as independent factors for differentiating LRTs from thymic cysts in our study (all *P* < .001). Notably, 87.50% (28/32) of LRT cases were located off-midline, compared to only 50% (20/40) of hyper-attenuating thymic cysts, consistent with previous studies.^[11,17]^ Both Ackman et al^[11]^ and Nam et al^[17]^ reported that thymomas tended to preferentially occupy an off-midline location, suggesting that this characteristic can serve as a basis for distinguishing between thymic cysts and LRTs. It is well established that the pathophysiological characteristics of thymic cysts and LRTs differ significantly; and CECT can effectively reflect the hemodynamic characteristics of these lesions.^[28,29]^ Our study demonstrated a statistically significant difference in enhancement degree: hyper-attenuating thymic cysts were more likely to exhibit mild to moderate enhancement (△CT < 25 HU), while LRTs were more likely to show moderate to severe enhancement (△CT ≥ 25 HU), aligning with previous reports.^[4,12,30,31]^

Most previous reports have primarily focused on individual CECT imaging features to differentiate TETs from thymic cysts.^[4,11,12,17,30]^ In contrast, our study is distinctive due to its comprehensive analysis of traditional CECT imaging features and clinical features concerning anterior mediastinal hyper-attenuating nodules, specifically LRTs and thymic cysts. We found that the AUC, sensitivity, specificity, and accuracy of the diagnostic model combining CTV, location, and enhancement degree of the hyper-attenuating nodules were 0.938, 0.719, 0.975, and 0.861, respectively. Although these traditional CECT imaging features exhibited relatively low specificity and accuracy for the diagnosis of LRTs when considered separately, their combined use in our study significantly enhanced the capability for differential diagnosis. Additionally, the simplified scoring system also demonstrated strong efficacy in differentiating LRTs from hyper-attenuating thymic cysts, achieving an AUC of 0.936, which was comparable to the diagnostic value of the predictive model. Importantly, the comparison of AUCs between the simplified scoring system and the predictive model (or nomogram) yielded no significant differences. When utilizing the nomogram to estimate the risk of LRTs, it is necessary to determine the points of each feature through visual comparison, which could easily result in inaccurate total scores and predicted percentages. In contrast, the simplified scoring system allows for the direct acquisition of scores for each feature without reliance on visual comparison. Therefore, due to its simplicity and convenience, the simplified scoring system has shown great potential in clinical practice.

It is important to note that our proposed model may offer treatment guidance for patients with anterior mediastinal hyper-attenuating nodules in clinical practice. Generally speaking, the higher the score obtained from the simplified scoring model, the greater the likelihood that the lesion was classified as LRTs. However, increasing the cutoff value may reduce sensitivity, potentially resulting in missed detections of LRTs, which could have serious consequences due to the biological uncertainty and invasiveness associated with such tumors.^[2]^ Conversely, lowering the cutoff value could reduce the likelihood of missed diagnosis, but it also decreased specificity, thereby increasing the risk of misidentifying hyper-attenuating thymic cysts as LRTs. This misclassification could lead to unnecessary psychological distress and surgical interventions.^[10]^ Therefore, we recommend thorough communication with patients prior to developing treatment plans because the selection of cutoff values reflects the level of risk that both physicians and patients are willing to accept.

Ultimately, the translation of artificial intelligence research into the optimization of clinical workflows is our primary objective. Our simplified scoring model is a valuable and convenient tool that can assist less experienced physicians or radiologists in situations where expert radiologists are absent or unavailable, particularly in resource-limited hospitals. Consequently, we believe this model may be an effective predictive tool for distinguishing between LRTs and thymic cysts, potentially reducing the need for unnecessary biopsies or surgeries.

Several limitations were identified in this study. Firstly, traditional CT imaging features may vary among radiologists due to the manual extraction process. Additionally, radiologists often lean towards diagnosing malignancy when faced with uncertainty, influenced by the likelihood of malignancy in real-world scenarios. Employing computer algorithms to measure such features could enhance the reproducibility and stability of the features and models. Secondly, our study was single-center and had a relatively small sample size. Future multicenter studies that encompass diverse racial groups and larger sample size may improve the predictive model’s performance to some extent. Thirdly, 2 CT scanners were utilized due to the retrospective nature of this study. Nonetheless, this can be viewed as an advantage of the study as it reflects real-world practice and offers potential generalizability.

In summary, the simplified scoring system, which was based on traditional CECT imaging features and clinical factors, demonstrated strong diagnostic performance in differentiating LRTs from hyper-attenuating thymic cysts. This system would assist clinicians in making informed pretreatment decisions. Furthermore, we believe that its convenience and simplicity make it highly suitable for widespread adoption.

## Author contributions

**Conceptualization:** Lin Zhao, Jingwu Li, Yongliang Liu, Wenzhe Zhao, Haifeng Cai, Lixiu Cao.

**Data curation:** Lin Zhao, Jingwu Li, Wenzhe Zhao.

**Formal analysis:** Lin Zhao, Jingwu Li, Wenzhe Zhao.

**Writing – original draft:** Lin Zhao, Jingwu Li, Wenzhe Zhao.

**Writing – review & editing:** Yongliang Liu, Haifeng Cai, Lixiu Cao.
